# Death by incarceration: Detention duration, overdose, and COVID-19 in Los Angeles County Jails, 2008–2023

**DOI:** 10.1371/journal.pone.0351332

**Published:** 2026-07-28

**Authors:** Adrianne Katrina Nelson, Michael Everett, Rachel Smith, Lindsey Rogers, Renee Grange, Rebekah Alfred, Haller Jackson, Lauren Woyczynski, Nikolas Brandt, Zainab Jamali, Muryam Hasan, Chanel Nejad, Ayesha Aslam-Mir, Josue Sanchez Hernandez, Abril Guanes, Diana Simmes, Aaron Littman, Sharon Dolovich, Nicholas Shapiro

**Affiliations:** 1 Yale Department of Health Policy and Management, School of Public Health, New Haven, Connecticut, United States of America; 2 Behind Bars Data Project, University of California, Los Angeles School of Law, Los Angeles, California, United States of America; 3 Carceral Ecologies, Institute of Society and Genetics, College of Life Sciences, University of California Los Angeles, Los Angeles, California, United States of America; 4 Department of Geography, College of Social Sciences, University of California Los Angeles, Los Angeles, California, United States of America; 5 Department of Sociology, College of Arts and Sciences, University of Washington, Seattle, Washington, United States of America; University of Foggia: Universita degli Studi di Foggia, ITALY

## Abstract

**Importance:**

Jail mortality in the United States has risen sharply in recent years, yet deaths in custody—especially those related to substance use—remain underexamined. The COVID-19 pandemic and the opioid crisis compounded pre-existing carceral health issues, necessitating a closer examination of their combined impact and aftermath. Previous reports suggest that incarcerated people—particularly those who overdose on drugs—die early in their confinement. This article indicates that the dual health threats of the COVID-19 pandemic and the opioid crisis have changed this relationship, substantially extending the time from arrest to death.

**Objective:**

To investigate how duration of confinement, manners and causes of death (e.g., COVID-19, substance use), and pandemic mitigation policies impacted mortality in Los Angeles (LA) County Jail from 2008–2023.

**Design:**

A retrospective, observational study utilizing linked datasets to identify mortality trends and associated factors.

**Setting:**

LA County Jail, the largest jail system in the United States.

**Participants:**

We created three data sets: 1) LA County Jail deaths from 2008–2023, N = 509, 2) City Jail deaths within LA County, encompassing LA City Jail deaths 2008–2023, N = 42 and Non-LA City Jail deaths, 2008–2023, N = 30, and 3) all bookings from 2010–2022, N = 1,435,479.

**Main outcome(s) and Measure(s):**

Mortality trends over time, duration of incarceration prior to death, duration of incarceration for those released or transferred, and cause-specific mortality including direct standardized rates and standardized mortality ratios. Statistical models assessed the impact of confinement duration and pandemic and opioid crisis years on mortality.

**Results:**

Jail mortality broadly, and substance-related deaths specifically, increased from 2008 to 2023, peaking during the pandemic. The median time from arrest to death was over five times as long (59 days) as the median duration of stay for all bookings (11 days). COVID-19 infection, prolonged confinement, and restrictive pandemic policies were associated with elevated mortality rates.

**Conclusions and Relevance:**

Findings highlight the urgent need for reforms, including reduced incarceration; reduced incarceration duration; expanded access to substance use disorder treatment; decreased influx of drugs into jails; increased programming, visits, and out-of-cell time; and improved health safeguards.

## Introduction

Public attention on deaths in custody often focuses on those killed during arrest. Yet in the United States, more people die every year in jails, most before they’ve gone to trial [1,2]. Despite known under-counting, national data indicates a sharp increase in jail mortality since 2019 [3]. This rise in death may be due to changing conditions and populations in U.S. jails, alongside the combined effects of the COVID-19 pandemic and the opioid epidemic [3].

From 2020 to 2023, the mental health and well-being of incarcerated persons suffered due to steps initially taken to slow the spread of COVID-19, including lockdowns, longer average stays in jail for those awaiting trial or transfer to prison, limited access to visitation, and increased use of “restrictive housing” or solitary confinement [4]. Qualitative research has illustrated how the pandemic deepened the “pains of imprisonment” via increased physical and emotional distress, a heightened sense of vulnerability, and increased mistrust of carceral institutions and their employees [5]. Additionally, from the start of the pandemic, access to consistent, in-person healthcare for all ailments declined for people held in jail [6] and sources point to a decline in primary and specialty care referrals, timeliness of care in California prisons, and mental health and drug treatment services in international carceral settings [6,7].

This decreased access to and lower quality of healthcare also impacted those seeking substance-use disorder treatment in jails [8]. Over the past two decades, drug- and alcohol-related deaths increased society-wide by over 360% [9]. This increase is largely due to increased use of fentanyl and other opioids from 2019 to 2022. Within this time period, opioid deaths increased by 100% nationwide, 121% in California, and 300% in California jails [10,11].

While substance-related deaths post-release are well documented in the literature, [12] relatively little research exists on substance-related deaths inside jails [8,11,13,14]. A 2021 systematic review found no documentation in the medical literature of any fentanyl-attributed overdose deaths in any correctional facility nationwide from 2013 to 2021, yet identified 76 fatal overdoses in media reports, more than three quarters of which occurred in jails [11]. An influential Bureau of Justice Statistics (BJS) report observed a median of one day from arrest to death for substance-related deaths in a national study of 1,742 jail deaths, implying that most drug overdose deaths occurred from substances imbibed prior to jailing [15]. We conducted oral histories with families impacted by jail deaths in Los Angeles to assess concerns within the impacted community that may not be captured in the existing literature or national-scale assessments and inform hypothesis generation. Findings pointed to substance-related deaths multiple years into incarceration [16].

Finally, exposure to incarceration itself has long been known to impair health and decrease longevity [17–19]. Incarcerated people have higher mortality rates, as well as elevated rates of infectious and chronic disease and mental illness, compared to the free-world population [19]. These disparities are due both to the disproportionate incarceration of lower-income populations with greater pre-existing health vulnerability and to the conditions of incarceration, which include poor access to healthcare, crowding, isolation from social networks, and mistreatment [18]. Just as the transition from incarceration back into society can produce acute health risks [20,21], so too can the transition into the jail system pose the simultaneous risks of interrupting continuities in health care and encountering harsh conditions [18]. However, it is unclear precisely which of the many unsafe conditions in jails, including those intensified during the COVID-19 pandemic, are exacerbating patterns of death and contributing to the recent increases in jail mortality.

In an effort to better understand the reported rise in jail deaths in Los Angeles (LA) County Jail, we consider: 1) the time from arrest to death in light of the population-wide duration of jail confinement; 2) all-cause, COVID-19, and substance-related mortality trends over time; 3) how much of the increase in mortality is independently attributable to the increase in the duration of confinement, substance use, or COVID-19 infection, versus the combined effect of those three things, together with other unmeasured variables, during the COVID-19 pandemic years of 2020–2022, and 4) how standardized mortality rates compare to those of other similar populations during the same period. We hypothesize that 1) contrary to common belief, the median time from arrest to death is longer than the median duration of confinement, and 2) the increased death toll in LA County Jail over the past 10–14 years is associated with substantially longer durations of incarceration and worse carceral conditions, particularly exposure to COVID-19 infection, COVID-19-related policy and practice changes, and increased opioid use.

## Methods

### Background

We focus this analysis on LA County, home to the largest jail system in the country [22]. In the first quarter of 2023, the LA County Jail had an average daily count of 14,253 people, a number greater than the total incarcerated populations of many states. Average daily count at the Los Angeles County Jail is down from about 17,000 in 2017 [23].

Between September 2022 and November 2024 we conducted in-depth oral histories with 14 family members impacted by jail deaths in LA County. Notes from the corresponding author, who conducted all interviews, served as the qualitative input for hypothesis generation.

### Data compilation

Our database includes mortality data from 2008−2023, compiled by the University of California Los Angeles (UCLA) Carceral Ecologies Lab through website scraping, 12 public records requests, and previously published data. Data from public records were obtained from documents produced by the LA Sheriff’s Department (LASD), the LA Department of the Medical Examiner (LADME), the California Department of Justice (CADOJ), and the LA County Office of the Inspector General (LACOIG). We used a web scraper to collect publicly available data from the LADME website and abstracted data from autopsies obtained via a pay-per-record fee. Information on deaths in LA County Jail was also linked to ICD-10-coded death certificates via the National Death Index (NDI).

Our decedent dataset consists of all 509 deaths occurring in LA County Jail from 2008–2023. Data includes first and last name, date of birth, race/ethnicity, sex, date of arrest, date of death, manner of death, place of death, and detailed cause of death, among other details. We linked records by name, date of birth, and date of death. LASD reported being unable to locate arrest records for some decedents (N = 9). In 496 cases, we were able to confirm the veracity of linked records. To ensure all deaths were accounted for, we further cross-referenced public records from repositories reflecting mandatory reporting of LA County Jail deaths—those operated by the CADOJ (Government Code § 12525), LADME (§ 27491), LACOIG (LASD policy 4–10/050.00)—and data abstracted from LASD public records created for compliance with the federal Death in Custody Reporting Act and published by Reuters [24]. In four cases we additionally consulted county cremation logs.

### Data cleaning

We created three data sets: 1) County Jail deaths 2) City Jail deaths 3) all bookings.

Our primary vital statistics data source for the “County Jail deaths” dataset was LADME, and was compiled from medical examiner and forensic investigator reports. When a date of death or manner of death was missing, we used data from LASD. For decedents with multiple bookings, we calculated time to death using the most recent arrest date. We included only people who had died after arriving at the jail, not those who died during arrest or in transport prior to booking.

Some individuals who are arrested by the Los Angeles Police Department (LAPD) and other municipal police departments within the county are held in small city jails for up to 72 hours before being booked into the County Jail. The largest of these small facilities is the LA City Jail. To account for this population, we compiled two separate datasets using data from the California Department of Justice and publicly available medical examiner data: “LA City Jail deaths” for deaths occurring in the LA City Jail during the same time period (2008–23; N = 42), and “Non-LA-City City Jail deaths” (2008–2023; N = 30) for the city jails within LA County and outside the City of LA (e.g., Long Beach, Burbank, Torrance, etc.) [10]. Because the “LA City Jail deaths” and “Non-LA-City City Jail deaths” datasets did not include arrest or booking date, we used Sequential Monte Carlo simulation to estimate time to death for each individual (range 0–72 hours) [25]. We ran a supplemental analysis to explore how including these deaths in the larger dataset would impact Table 1 time to death comparisons (see S1 File).

**Table 1:** Characteristics of individuals deceased in jail by days from arrest to death, N = 509.

	N (%) or mean [SD]	Mean days	Statistic (p-value)	Median days	Statistic (p-value)
**Gender**					
Female	40 (8%)	91.69	**12.73 (0.0004)**	16.00	**5754 (0.0004)**
Male	460 (90%)	261.41		63.00	
Missing	9 (2%)	107.86		57.00	
**Race** ^*^					
Latine	195 (38%)	310.59	**2.94 (0.01)**	63.00	**16.67 (0.005)**
Black	151 (30%)	232.68		57.00	
White	126 (25%)	138.11		43.00	
Asian	17 (3%)	324.81		113.00	
Indigenous/ Pacific Islander	4 (1%)	26.00		38.00	
Unknown/ Other	5 (1%)	921.20		215.00	
Missing	11 (2%)	133.67		142.00	
**Age at death**	46.20 [15.28]				
18-30	106 (21%)	238.88	0.092 (0.97)	59.50	4.09 (0.25)
31-44	120 (24%)	268.95		40.00	
45-54	122 (24%)	231.53		55.00	
55 and over	157 (31%)	247.56		80.00	
Missing	4 (<1%)	80.67		51.00	
**Custody status**					
Unconvicted	340 (67%)	205.12	0.60 (0.44)	44.50	**28983 (<0.0001)**
Convicted	138 (27%)	235.48		121.50	
Missing	31 (6%)	1141.94		54.0	
**Death place**					
Jail	214 (42%)	241.97	0.16 (0.85)	43.50	**6.88 (0.03)**
Hospital	269 (53%)	256.47		69.50	
Other	2 (<1%)	45.50		45.50	
Missing	24 (5%)	173.95		50.00	
**Cause of death**					
Natural	265 (52%)	237.57	1.4 (0.22)	66.50	**15.20 (0.004)**
Accident	106 (21%)	345.65		54.00	
Suicide	76 (15%)	189.52		29.00	
Homicide	27 (5%)	273.28		127.00	
Undetermined	31 (6%)	113.23		36.00	
Missing	4 (1%)	80.67		51.00	
**COVID-rel. death**					
Yes	21 (4%)	452.67	2.68 (0.10)	160.00	**2602 (0.0004)**
No	472 (93%)	241.53		55.00	
Missing	16 (3%)	87.73		49.00	
**Acute substance-related death**					
Yes	66 (13%)	475.00	**11.84 (0.0006)**	63.00	13822 (0.79)
No	441 (87%)	212.71		56.00	
Missing	2 (<1%)	51.00		51.00	
**Substance-related type** ^†^					
Acute (overdose)	66 (13%)	475.00	**20.05** **(<0.0001)**	63.00	13268 (0.79)
Chronic	40 (8%)	168.25		33.00	
Potentially chronic	2 (<1%)	417.00		417.00	
Undetermined	14 (3%)	118.86		43.50	
Traumatic w/ substances	4 (1%)	81.33		51.00	
Adverse effects of drugs	2 (<1%)	83.00		83.00	
Not substance-related	379 (74%)	221.76		64.00	
Missing	2 (<1%)	51.00		51.00	

Missing values excluded in statistical tests

* Variables collapsed into Black, Latine, White, Other for statistical tests

†Variables collapsed into substance-related and not substance-related for statistical tests

Finally, we created a third dataset, “all bookings” of all people booked into LA County Jail, as the best available proxy for those who survived their jail time and were released or transferred out (typically to prison). This dataset combined yearly roster data provided by LASD, the California Board of State and Community Corrections, and the public records obtained and published by UCLA Million Dollar Hoods project (N = 1,133,352). These data were only available from 2010–2022. We removed duplicate observations traceable to a single person having multiple charges or being arrested multiple times in one year. There were thousands of individuals who had not yet been released from jail at the time of data issuance (September 15, 2022) and were therefore missing release dates. Of those booked in 2020, 558 had yet to be released; for 2021, 1,402 people; and for 2022, 6,735 people. To calculate median days from booking to release without error introduced by partial right-censoring, we only include booking dates until July 2022, which is >99% of bookings.

The final dataset included 1,435,479 observations. Because the “all bookings” dataset included a booking date but was partially missing the arrest date, we imputed missing arrest dates based on the non-missing arrest and booking dates (n = 133,352). In that dataset, the difference between arrest and booking dates was minute. The median, and 1st and 3rd quartiles were zero and the mean difference was 0.16 days. After multiple computational and manual attempts, we were unable to remove jail deaths from the jail roster used for the all-bookings dataset. Although decedents are themselves reflected in the bookings data, the potential impact on our findings is extremely minimal, since their bookings comprise far less than one-tenth of one percent of total observations.

Using medical examiner-determined cause and manner of death, we labeled each case within one of the following categories related to substance use: acute, chronic, potentially chronic, adverse effects of drugs, traumatic death with substance use, undetermined, and not applicable. These categories align with the CDC standard for reporting a cause of death as due to drug or alcohol consumption [26]. We then added the “potentially chronic” category so as to include two cases that, in the medical examiner’s determination, were not robustly classifiable as chronic due to language of “probable” chronic effects or “probable narcotics use”. To create a variable that captures deaths attributable to the COVID-19 virus, we labeled all cases with COVID-19 as a cause of death (n = 16) or those that mentioned the infection among other conditions contributing to but not directly related to the immediate cause of death (n = 5). All cases in the former category died of mechanisms known to be exacerbated or caused by COVID-19 infections [27–30]. Cause and manner of death data were accessed for research purposes on May 9, 2024, and are retained by the co-authors.

### Statistical analysis

We conducted all statistical analyses using R. We used the chi-squared test for binary categorical variables and Pearson’s chi-squared test to detect differences in sets for categorical data with greater than two categories. Due to non-normal distribution of the continuous variable in Table 1, we used the Mann-Whitney U test (two groups) and the Kruskal Wallis (three or more groups) to detect differences in medians and the Mood test with ordinal exposure variables. The one-way ANOVA was used for mean testing. Given the skewness of the data, the one-way ANOVA functioned as a secondary sensitivity test. Pearson’s correlation or Ordinary Least Squares (OLS) regression was used to estimate parameters in the linear regression model.

Because we were interested in how time-specific events such as the COVID-19 pandemic impact cause-specific and non-cause specific mortality, we chose to model trends over time rather than taking a cross-sectional approach. For crude mortality rates, we used the monthly average daily inmate population (ADIP) from LASD. We tested for but found no significant change in age demographics over time, so we used crude mortality without adjusting for age. To identify changes in mortality rate over time, we individually regressed monthly all-cause mortality rates and monthly substance-related mortality rates over time. Because COVID-19 deaths were limited to a two-year period, we did not conduct a regression for these deaths over time. To further understand how the pandemic years may have impacted the change in mortality rate over time, we ran a multiple regression model including as co-variates duration of confinement, COVID-19 deaths, and substance-related deaths. We then created a binary variable for the pandemic years (2020–2022) and included it in the model. Finally, to see if associations held, we tested the models for the pre-pandemic years of 2010–2018 and the post-Realignment years 2012–2018 (at which point California, to reduce prison overcrowding, began requiring some people to serve their prison sentences in county jail). We tested assumptions for linearity and homoscedasticity by plotting residuals versus fitted values for the regression model.

We chose not to include LA City Jail deaths in our primary analysis. This choice limits comparability to other major U.S. population centers like Chicago and New York that, at the behest of legislators, merged city and county jail functions into single unified authorities. Yet, the separation of city and county jails is commonplace throughout much of the South, West, Midwest, and, to a lesser extent, the Northeast. Retaining the scope of the study to match this common governmental structure additionally aligns with how in-custody death data is submitted to local, state, and federal oversight bodies such as BJS. Other data integrity issues motivating this approach include: 1) LASD maintains the population denominator data required for mortality rate construction that is not provided or reliable for LA City Jails 2) LASD’s unified administrative structure ensures consistent reporting throughout the study period whereas municipal jails likely employ heterogeneous custody classification practices and potentially introduce bias into trend models (See S1 File).

In both the LA County and LA City Jail datasets we include all deaths that the corresponding agencies delineate as jail deaths, including jail deaths labeled as “pre-booking.” To account for potential collection bias related to excluding individuals who died in the primary City Jails before being booked into the County Jail, we re-ran all analyses for Table 1 using a merged dataset with both “City Jail” and “County Jail” deaths. This dataset included 72 additional deaths during the study period (See S1 File).

We compared the proportions of deaths and all bookings within each demographic category using two-proportion z-tests. To account for multiple comparisons, p-values were adjusted with the Holm correction, and categories with adjusted p < 0.05 were considered significantly different.

To categorize cause-specific mortality patterns within the Los Angeles County Jail, deaths were first classified by ICD-10 underlying cause codes from NDI death certificate data reviewed with context from medical examiner reports using the categories outlined in the Cook County Jail 10-Year Report (1995−2004) [31]. ICD-10 codes for underlying COD were grouped into the following categories: illness deaths (e.g., heart diseases [I00-I09, I20-I25, I27-I51], malignant neoplasms [C00-C97], cerebrovascular diseases [I60-I69], chronic respiratory diseases [J40-J47, J60-J67], diabetes [E10-E14], liver diseases [K70-K76], hypertension [I10, I12, I15], aortic aneurysm [I71], pulmonary embolism [I26], gastrointestinal bleeding [K25-K28, K62.5, K92.0-K92.2]), infectious and inflammatory deaths (e.g., pneumonia [J12-J18], septicemia [A40-A41], HIV [B20-B24], meningitis [A39, G00-G03]), and non-illness or external causes (unintentional injury [V01-X39, X45-X59, Y85-Y86], suicide [X65-X84, Y87.0], drug overdose or withdrawal [X40-X44, X60-X64, Y10-Y14], homicide [X85-Y09, Y87.1], and ruptured ectopic pregnancy [O00]).

Data for deaths in California and the U.S. were gathered from the CDC WONDER system and the National Vital Statistics Service Multiple Cause of Death system. We calculated both directly and indirectly standardized mortality metrics based on this cause-specific data from 2011–2022. Directly standardized rates (DSRs) were computed by applying age-specific mortality rates from the jail population to the U.S. general population in 2000 to estimate the rate per 100,000 if the jail and U.S. and California populations shared the same age structure. Standardized mortality ratios (SMRs) were derived via indirect standardization, applying U.S. and California age-specific rates to the jail population to compute expected deaths and dividing observed by expected counts. Confidence intervals were estimated using exact Poisson limits. All rates were standardized only for populations aged 15 or older to reflect the age structure of the jail system.

Individual-level LA County Jail booking data yielded a population that was on average 18% lower than LA Sheriff data on annual ADIP. As a result, we took a conservative approach by utilizing the age distribution of the aggregated individual booking data and applied it to the ADIP for each year to reconstruct demographics for SMRs. ADIP is also the population data source most used in county and state reporting and policy discussions. Because the DSR reflects an absolute rate standardized to an external population and the SMR expresses a relative excess mortality weighted by the jail’s own age structure, the two measures need not align and may emphasize differences in the age-structure of the jail system compared with the U.S. and California populations.

### Ethics approval

National Death Index (NDI) data were analyzed under the UCLA Office of the Human Research Protection Program 01/05/2024 (IRB#24–002406). Other data analysis in this study in the form of 1) exploratory oral histories 7/26/2022 (IRB#22–001219) and 2) additional quantitative data analysis 08/25/2023 (IRB#23–001093) was deemed exempt by the UCLA Office of the Human Research Protection Program.

### Terminology

We use the term Latine to refer to people of Latin American or Caribbean origin or descent, following gender-inclusive conventions increasingly used by Spanish-speaking communities, rather than Latino/a/x or Hispanic. All other racial/ethnic categories used in this article follow standard classifications.

## Results

In the LA County Jail, we found that deaths occur well into the jail stay (Table 1). From 2008 to 2023, the median duration from arrest to death was 57.5 days. Only 21 (4.1%) of deaths occurred within one day of arrest, 85 (16.7%) within one week, and 124 (24.4%) within two weeks. For the years 2010−2022, for which we have release/transfer dates for those in the jail population (described herein as duration of stay), the median time from arrest to death was over five times longer (59 days) than the median duration of stay (11 days). COVID-19 (median), acute substance-related deaths (mean), and homicides (median) had significantly longer times to death compared to other causes of death.

When compared to the total booked population, the county deceased population was more consistently male (91% versus 83%). Latines were the largest racial group in both populations, followed by Black, White, and Asian: 39% Latine decedents versus 51% Latine of the total population booked, 30% Black decedents versus 28% booked, 26% White decedents versus 18% booked, and 6% belonging to other races versus 4% belonging to other races in the total booked population. Decedent distribution was markedly older than the all-bookings population, which contained almost half (45%) under 30 versus 23% among decedents, 34% 31–44 years versus 22% among decedents, 14% 45–54 years old versus 23% among decedents, and 6% over 55 years, versus 32% among decedents. The youngest decedent was 18 years (n = 2) and the oldest was 86 years (n = 1).

Table 2 displays specific comparisons among demographic factors between deaths and bookings in the LA County Jail. The results show significantly higher representation of older age groups, males, and white and ‘other’ race populations in deaths as a proportion of all bookings. There was lower representation for younger age groups, women, and Latine population.

**Table 2:** Demographic proportions of deaths compared to all bookings, 2010-2022 (N = 397).

	Deaths (n)	All Bookings (n)	Statistic (p-value)	Proportion of Deaths	Proportion of All Bookings
55-100	128	90978	*p < 0.001*	0.32	0.06
45-54	90	205679	*p < 0.001*	0.23	0.14
31-44	87	489142	*p < 0.001*	0.22	0.34
18-30	89	649668	*p < 0.001*	0.23	0.45
Male	355	1184368	*p < 0.001*	0.91	0.83
Female	34	251110	*p < 0.001*	0.09	0.17
Latine	151	728002	*p < 0.001*	0.39	0.51
Black	115	400786	0.465	0.30	0.28
White	99	251742	*p < 0.001*	0.26	0.18
Other	22	54949	*p =0.153*	0.06	0.04

**Fig 1:**
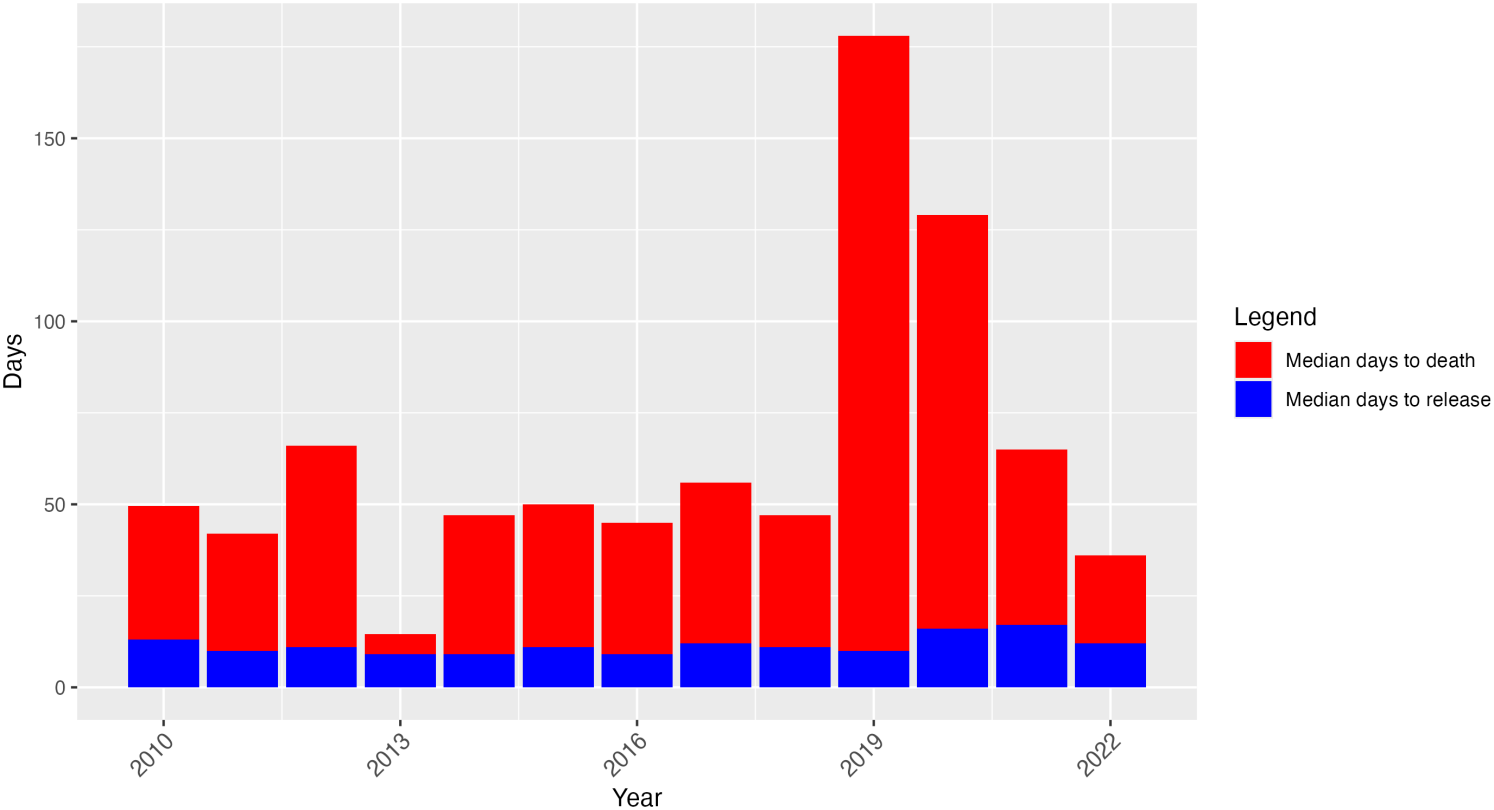
Median days to death and duration of confinement in LA County Jail, 2010-July 2022.

**Fig 2:**
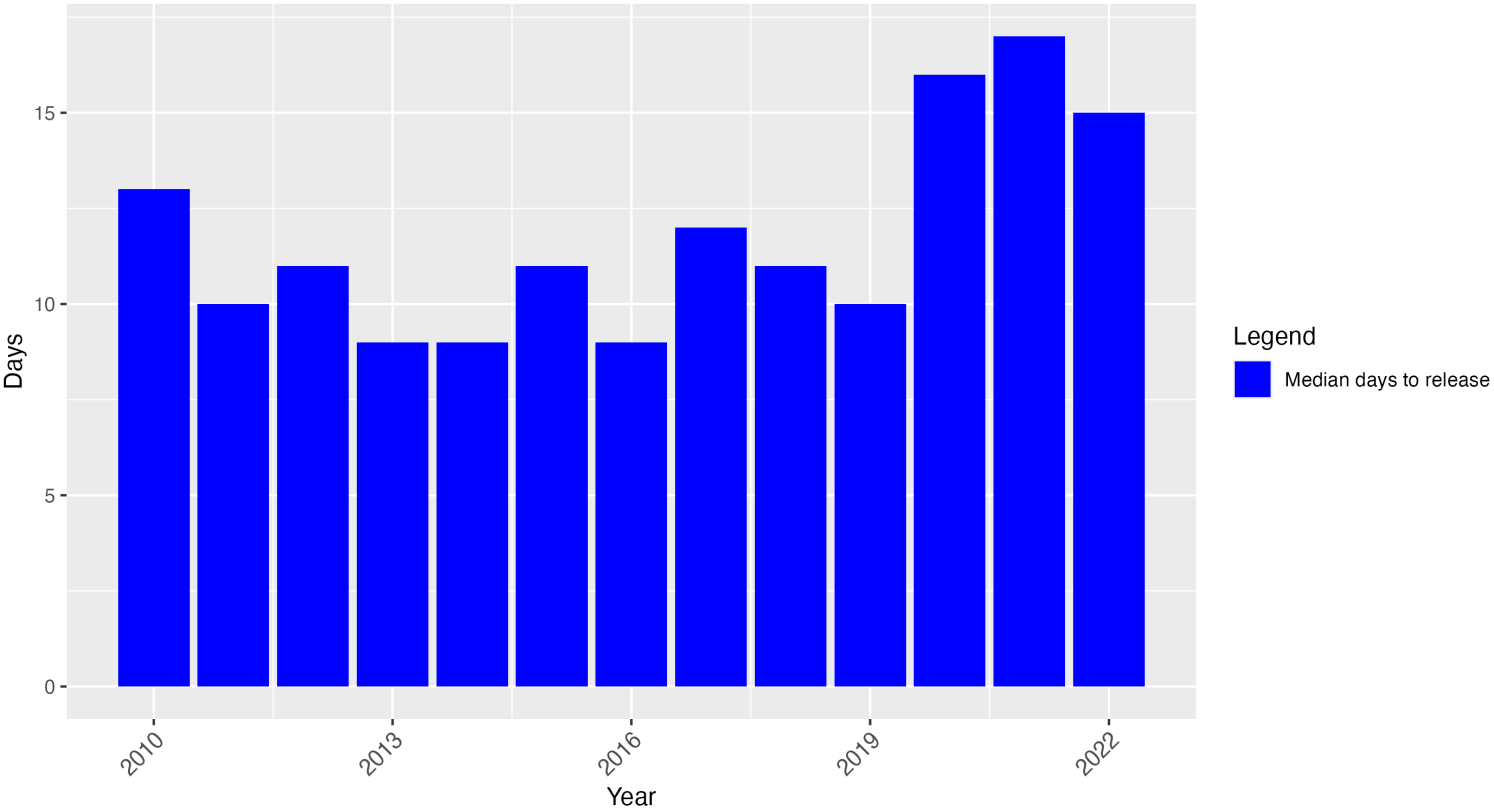
Median duration of confinement in LA County Jail, 2010-July 2022.

The increase in time from arrest to death from 2008 to 2023 is significant (Fig 1*, Corr = 0.36, p <0.001)*, as is the increase in duration of confinement for all bookings from 2010 to 2022 (Fig 2*, Corr = 0.47, p < 0.001)*. The latter association loses significance for the pre-COVID period of 2010–2018 *(Corr = -0.04, p = 0.65)* but remains significant for the post-Realignment years of 2012–2018 *(Corr = 0.31, p = 0.003) independently.* Duration of confinement for both those who survive and those who die in LA Jails has increased and periods of policy change (Realignment + COVID) appear to contribute to these increases.

**Fig 3:**
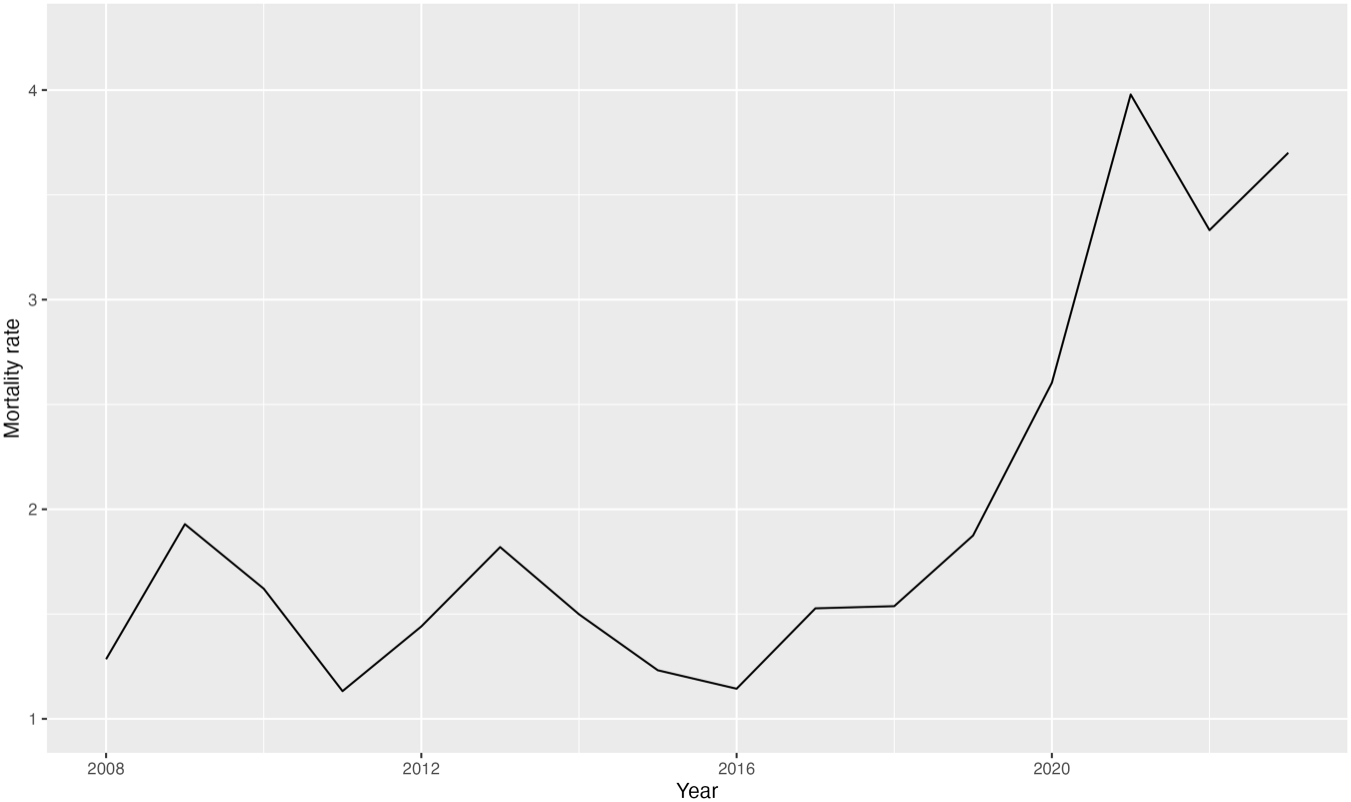
Crude mortality rate per 1000 by year in the LA County Jail, 2008-2023.

Following the date range of available bookings data, the crude monthly mortality rate in the Los Angeles County Jail increased significantly from 1.62/1000 in 2010 to 3.33/1000 in 2022, peaking at 3.98 in 2021 (Fig 3*, Corr = 0.72, p = 0.001)*. Mortality did not significantly increase during the pre-COVID *(Corr = −0.07, p = 0.87)* or post-Realignment years *(Corr = −0.22, p = 0.625).*

**Fig 4:**
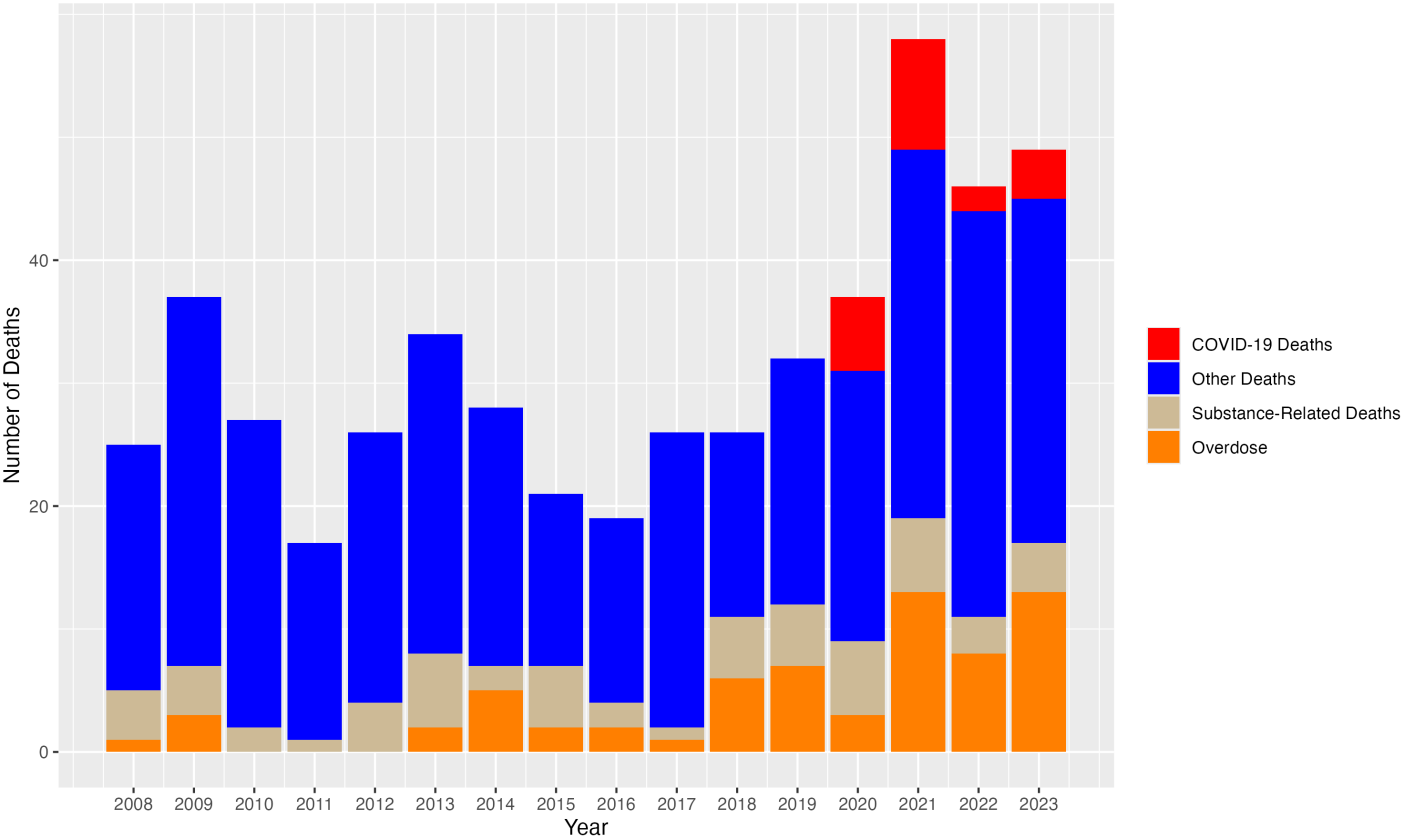
Proportion of deaths in the LA County Jail accounted for by COVID-19 infection and acute substance use by year, 2008-2023.

The number of substance-related deaths, and substance-related deaths as a proportion of total population, increased significantly from 2008 to 2023 (Fig 4*, R*^*2*^ *= 0.24, p < 0.0001 and R*^*2*^ *= 0.27, p < 0.0001).* Likewise, acute substance-related deaths (overdoses) (*R*^*2*^ *= 0.22, p < 0.0001*), and acute substance-related deaths as a proportion of total population, increased significantly from 2008 to 2023 (*R*^*2*^ *= 0.23, p < 0.0001*), accounting for only 4% of total deaths in 2008 and almost 26.5% of deaths in 2023.

In linear multiple regression modeling, mortality still increased significantly from 2010 to 2022 when controlling for duration of confinement *(R*^*2*^ *= 0.37, p < 0.0001),* and when removing just deaths attributed directly and indirectly to COVID-19 infection from the sample *(R*^*2*^ *= 0.26, p < 0.0001).* When removing just the substance-related deaths, the increase in mortality was also significant *(R*^*2*^ *< 0.18, p =.01)*. However, when both COVID-19 and substance-related deaths were removed from the model (accounting for duration of confinement), it became insignificant (*R*^*2*^ *= 0.14, p = 0.07)*, suggesting the increase in mortality from 2010 to 2022 is independently associated with a steep rise in deaths from the COVID-19 pandemic and the opioid epidemic. Likewise, the same model including only the pre-COVID years of 2010–2019 found an insignificant rise in the crude mortality rates in linear modeling when controlling for duration of confinement *(R*^*2*^ *< 0.1, p = .4).*

In our analysis of age-adjusted cause-specific mortality covering 2011−2022, the LA County Jail all-cause Directly Standardized Rate (DSR) is 709 per 100,000 (95% CI 494−925). Standardized mortality ratios (SMR) are significant and suggest higher mortality risk among the LA County Jail population compared to the U.S. general population deaths for pulmonary embolism, GI bleeding, pneumonia, septicemia, suicide, and heart disease (Table 3). In contrast, the risk of mortality from chronic respiratory disease, unintentional injuries, malignant neoplasms, diabetes, liver diseases, and all cause mortality are significantly lower in the Jail than in the U.S. general population.

**Table 3:** SMR comparisons for LA County Jail vs. California vs. U.S. general population, 2011-2022.

Cause of death	LA County Jail DSR	US DSR	California DSR	LA County Jail – US SMR	LA County Jail – California SMR	California – US SMR
All cause	709.49	952.27	783.60	0.8**	1.13*	0.82**
Aortic aneurysm†	2.47	3.31	2.45	2.03	2.72	0.74**
Cerebrovascular diseases	41.32	49.11	48.15	1.07	1.28	0.98**
Chronic respiratory diseases†	5.31	50.10	40.01	0.25**	0.41	0.79**
Diabetes†	6.4	28.11	27.58	0.36*	0.45*	0.98**
Drug overdose or withdrawal	24.45	26.38	18.63	0.95	1.49**	0.73**
GI bleeding†	3.55	4.20	2.20	3.79*	9.82**	0.53**
HIV†	0.91	2.12	1.35	0.58	1.04	0.66**
Heart diseases	177.43	195.27	169.68	1.34*	1.86**	0.87**
Homicide	12.41	8.01	–	0.92	–	–
Hypertension†	0	11.85	16.61	0	0*	1.4**
Liver diseases†	6.59	18.71	18.20	0.43*	0.44*	0.98**
Malignant neoplasms	69.05	194.18	168.35	0.27**	0.35**	0.87**
Meningitis†	0	0.19	0.06	0	0	0.56**
Pneumonia	44.41	16.35	16.60	2.55**	3.63**	1.21**
Pulmonary embolism	10.10	2.93	1.27	5**	12.09**	0.44**
Ruptured ectopic pregnancy†	0	0.01	–	0	–	–
Septicemia	42.87	13.13	4.28	2.18*	7.28**	0.33**
Suicide	88.68	17.02	10.85	1.65**	2.72**	0.73**
Unintentional injury (accidents)	10.07	37.55	21.51	0.25**	0.43**	0.60**

** p < 0.01, * p < 0.05, † fewer than 10 deaths in the LA County Jail. Homicide-related deaths for California in the CDC Wonder system do not appear to be comprehensive and no counts were produced for ruptured ectopic pregnancy mortality, likely due to low incidence in California.

Standardized mortality ratios were higher when comparing LA County Jail to California than to the U.S. general population, reflecting California’s generally lower baseline mortality rate. Notably, while there was no greater mortality risk in the LA County Jail compared to the U.S. general population for drug overdoses/withdrawal deaths (0.95, 95% CI 0.72–1.24, p > 0.05), there was greater mortality risk for this cause of death in the LA County Jail when compared to California (1.49, 95% CI 1.13–1.95, p < 0.01). All-cause mortality risk for the LA County Jail is significantly elevated when compared to California (1.13, 95% CI 1.02–1.25, p < 0.05).

## Discussion

### Length of stay

In LA County, we found that jailed people die much further into their stay than reported by BJS. In a report spanning 2000–2019, BJS found that 40% of jail deaths nationwide were within one week of incarceration, as compared with 16.7% of our sample from 2008 to 2023. BJS reported a median of 17 days to death post-arrest, versus 57.5 days in our sample [15]. Additionally, according to the BJS report, substance-related deaths had the shortest time to death (median of one day, N = 1,742), suggesting that when incarcerated people die of overdose or withdrawal in jail, they do so almost immediately after booking. However, we found that the median days from arrest to death for acute substance-related deaths (63 days) in LA County was over 60 times higher than that reported by BJS [15]. This discrepancy raises the question whether the LA County result is an outlier or this local study is a harbinger of a national shift in jail mortality patterns since the last BJS report. Further questions arise about the completeness and integrity of data reported by local, state, and federal agencies upon which the BJS study was based.

Our findings suggest that incarcerated people are gaining access to opioids and, we speculate, using them while jailed as a means of enduring dismal conditions and extended periods of confinement. Given the sharp upward trend in substance-related deaths we report, those committed to reducing jail deaths will want to prioritize improving access to meaningful substance-use disorder treatment and opioid agonists; improving conditions for those inside, including access to other healthcare; and undertaking more effective means for preventing deadly substances from getting into the jail. High rates of acute substance use death during periods when pandemic protocols curtailed family visitation indicates the need for scrutiny of staff ingress. Our finding that 70% of jail deaths occur pretrial (excluding those missing custody status) further emphasizes the lethal consequences of incarceration prior to adjudicating guilt.

### *COVID-19, pandemic policies, and the* opioid *epidemic*

Our findings support research showing that there was elevated risk of death during the pandemic years for incarcerated people even beyond the risk posed by COVID-19 infection and by increased availability of powerful opiates [32,33]. Mortality, which was not on the rise pre-pandemic, increased significantly during the pandemic years, even when controlling for duration of confinement, COVID-19, and acute substance-related deaths. We believe that a mix of criminal justice policies and procedures, resulting in slowdowns in court processing, decreased access to healthcare including mental health and substance use treatment, increased use of restrictive housing, decreased staffing, and decreased visitation rights may be responsible for the increased lethality of this large jail system during the pandemic. These policies and procedures made the experience of incarceration harsher and increased the time incarcerated people were forced to endure it, despite multiple efforts to reduce the jail population at the start of the pandemic and the consequent 5,000 person decline in average daily jail population by June of 2020 [34]. These deaths call for a deeper examination into how procedures and policies in carceral settings, especially those that impact the quality and duration of incarceration, shorten lives. Our findings support existing in-custody health priority areas (reduce incarceration, expand substance use intake assessment and treatment, reduce influx of illicit substances, increase efficacy in health care management, increase access to visits, programming, and out-of-cell time) [35–40] while also indicating the importance of decreasing the duration of jail time.

### Standardized mortality ratios

We observe similar trends to those reported in the Cook County Jail from an earlier period (1995–2004), finding that the LA County Jail had significant positive SMRs for pneumonia, septicemia, and heart diseases compared to the U.S. general population [30]. The LA County Jail also maintained significantly lower SMRs for chronic respiratory diseases, malignant neoplasms, diabetes, and unintentional injury. Causes of death that substantially increased in risk between our study and the Cook County Jail study included aortic aneurysm, cerebrovascular diseases, GI bleeding, pneumonia, pulmonary embolism, septicemia, and suicide. While all-cause mortality was significantly lower in the Cook County Jail compared with the U.S. population from 1995–2004, we find no difference between mortality in the LA Jail and in the U.S. population from 2011–2022, suggesting geographic and/or temporal differences in jail mortality over time.

We additionally compared the LA Jail population to the California population as a more geographically proximate reference population may be a more appropriate benchmark for evaluating health inequalities. Comparing the LA Jail System to the California population yielded generally higher SMRs than the national comparison, including for all-cause mortality. For example, the LA Jail’s suicide SMR was higher relative to California than relative to the U.S. Additionally, while the LA Jail’s all-cause SMR was lower relative to the U.S. general population, it was higher relative to California.

If our findings for LA County mirror trends in other U.S. jails, the comparative risk of mortality in U.S. jails has likely increased compared with that experienced by the general population in recent years. This change in risk is due to some combination of improved mortality outcomes for the general population and increased risk of mortality for certain conditions in jails. With a few exceptions, like HIV and meningitis, the protective effect of incarceration for conditions found in the 1990s-2000s has diminished. The risk to jail detainees of other potentially preventable causes of death like suicide, pneumonia, septicemia, and pulmonary embolism has also increased compared to the prior study.

The highest consistent SMR, pulmonary embolism, aligns with a recent autopsy-verified analysis of deaths in the LA County Jail, suggesting that reduced freedom of movement, restraints, physical trauma, and high psychotropic prescription rates might account for a significantly higher rate of venous thromboembolism death compared to the general county population [41]. Protective SMRs for chronic conditions such as diabetes and respiratory disease indicate the capacity for carceral health systems to mitigate health risks for other illnesses that have traditionally been significant drivers of mortality in incarcerated populations.

### Limitations

While we do not have data to test the impact on mortality of specific policies or procedures adopted during the COVID-19 pandemic years, these findings present initial evidence that a combination of increased availability and potency of opiates, COVID-19 infections, and policies and procedures implemented during the pandemic which greatly restricted movement and social interaction may have contributed to the dramatic spike in LA County Jail deaths from 2020–2023. Although we are unable to fully demonstrate here the causal impact of all the policy recommendations we propose, the value of each is suggested by our findings and supported in the broader literature on carceral mortality as likely mechanisms for reducing mortality in carceral settings.

The data needed to conduct mortality studies in carceral facilities are becoming even more difficult to obtain. Even basic data, such as that reported prior to 2019 by the United States Department of Justice, are no longer being released publicly. Researchers therefore depend on a patchwork of state laws and the cooperation of corrections officials and medical examiners to obtain data through public records requests. Such data acquisition can be slow and involve redaction and discrepancies between sources. Information about substance-involved cases is additionally difficult to obtain as reports are often withheld for long periods of time due to toxicology backlogs and are subject to heavy redaction even when released [14].

In order to include as many observations as possible given available data and without impacting the validity of the dataset, we identified July 2022 as a date that would allow us to include 99.5% of the sample. This excluded percentage diminishes the farther back the dataset goes and the more complete the release data is. We do not think this dynamic significantly impairs validity of the findings, because the dataset in its entirety includes over 1.4 million observations and spans 12 years.

We cannot confirm certain details of substance-related deaths, such as the substances involved, or determine whether any deaths were misclassified as non-substance-related, or whether any withdrawals were misclassified as acute events (overdoses). One case declared an overdose by the medical examiner has been contested by the family, which asserts that the death was due to acute withdrawal and not to overdose [42]. Data collected from bookings records may also have contained inaccuracies due to individuals using different aliases or dates of birth or issues in the jail record system. Finally, we used ADIP data for year-by-year population size. This population was consistently larger, and we suspect more complete, than the population represented in bookings data. Using the larger population may result in underestimation of SMRs.

## Conclusion

Our data suggest that a “perfect storm” of fatal conditions occurred in the LA County Jail during the pandemic years—namely, a combination of increased fatal access to controlled substances, COVID-19 infection and sequelae, and policies and practices further intensifying the harshness of detainees’ daily lives, all of which contributed to a ballooning effect on mortality. Additionally, across all years, people are dying during stays much longer than the median stays of all those booked into the jails. Now that the pandemic has officially ended, it remains unclear how carceral policy will curtail or perpetuate longstanding health hazards that have been magnified in recent years. In 2025, 46 deaths were posted in LA County Jail–higher than any year in our sample other than 2021–indicating that the drivers of mortality that intensified in 2020 have not simply ebbed as the pandemic receded [43].

## Supporting information

S1 File Incarceration durations, LA County vs City Jail deaths. (DOCX)
