# Supplementary material for: Death by incarceration: Detention duration, overdose, and COVID-19 in Los Angeles County Jails, 2008–2023
Source: PLoS One. 2026 Jul 28;21(7):e0351332. doi: 10.1371/journal.pone.0351332 (PMC13411872; doi:10.1371/journal.pone.0351332)
Supplement: S1 File — (DOCX) [file pone.0351332.s001.docx]

### S1 An incarceration duration comparison of LA County Jail deaths to LA City Jail deaths, 2008-2023

In addition to the 509 Los Angeles County Jail deaths analyzed in this study, deaths in 16 different city jail systems (there are 88 incorporated cities within LA County) were documented by the California Department of Justice during the study period, amounting to 72 additional jail deaths. These individuals were temporarily held in city jails per the California Penal Code, which requires arraignment or release within 48 hours of arrest in most cases. Those arrestees from city jails who remain in custody after arraignment, with some exceptions such as those with federal judicial warrants, are remanded to LASD jail custody.

In order to explore how these city jail deaths would impact our analysis, we added them to the larger LA County Jail deaths dataset and ran tests of association. As these deaths are less than 3 days, mean and median durations from arrest to death decreased and the percentage of pre-trial deaths increased. Inclusion of city jail deaths changed the statistical significance of two median subgroup comparisons: age at death and substance-related death type (Supple. Table 1).

We chose to restrict our analysis to LASD facilities for multiple reasons: 1) Assembling jail death and AIDP datasets as robust as our LASD datasets for the city jails would have likely entailed writing more than 100 additional public records requests, which was not feasible for this study. LASD represents the overwhelming majority of the county jail population and maintains a well-documented monthly ADIP denominator necessary for mortality rate construction. LAPD, in contrast, does not publicly provide ADIP and arrest records requested by other researchers has yielded release date missingness of 30%.[^1^](https://www.zotero.org/google-docs/?Zb2XGQ) 2) LASD operates under a unified administrative and reporting structure across the full study period. Municipal jail facilities likely vary in reporting practices. Of the jail deaths for which we have nuanced custody status, 1% (5 of 489) of LASD jail deaths are labeled as “awaiting booking” whereas 16.7% (12/72) of city jail deaths are labeled “awaiting booking”. This suggests different institutional practices of delineating between “process of arrest deaths” versus “jail deaths” which would preclude construction of comparable mortality rates and potentially introduce heteroscedasticity into trend models. Taken together, these differences in administrative classification and data availability suggest that the city jail deaths, while valuable for context, do not meet the methodological standards required for inclusion in the primary analysis.

When we added the LA City Jail deaths to the LA County Jail deaths median significance changed for two variables in the length of stay analysis. 1) Age: median days to death decreased across all age groups as would be predicted a priori. With the added city jail deaths, age became significant (*p*=0.97 → p=0.03) driven by a near halving in median time from arrest to death for the 31–44 age group (40 → 22 days). This is the modal age group (53.3%) for city jail death suicides, a major contributor to the shortening of median durations of arrest to death. 2) Substance-related Type: Median days from arrest to death for the type of substance-related death became significant. When adding city jail deaths into the dataset, many more short-term substance-related deaths were added, dramatically reducing the median days from arrest to death.

Acute substance use death incarceration durations dropped nearly three quarters. Despite this drop, the median remains 18-times higher than the national median days from arrest to death as reported nationally by the U.S. Department of Justice.[^2^](https://www.zotero.org/google-docs/?UHFz6v) Chronic substance use death incarceration durations dropped by two thirds. The changes add nuance to the findings presented in the main manuscript without substantially altering them. These insights are likely to be fruitful grounds for further research that develops a clearer picture of contextual aspects and population outcomes of municipal jail confinement.

**Supple. Table 1. Characteristics of individuals deceased in jail by days from arrest to death including city deaths with Monte-Carlo simulation, N=581**

|  | **N (%) or mean [SD]** | **Mean days** | **Statistic (p-value)** | **Median days** | **Statistic (p-value)** |
| --- | --- | --- | --- | --- | --- |
| **Gender** |  | | | | |
| Female | 50 (9%) | 73.32 | **3.74 (0.05)** | 6.00 | **8343 (0.0001)** |
| Male | 522 (90%) | 229.80 |  | 43.50 |  |
| Missing | 9 (1%) | 107.86 |  | 57.0 |  |
| **Race**^*^ | | | | | |
| Latine | 222 (38%) | 271.92 | **3.02 (0.03)** | 42.50 | **28.07 (<0.0001)** |
| Black | 161 (28%) | 218.15 |  | 57.00 |  |
| White | 154 (27%) | 112.79 |  | 43.00 |  |
| Asian | 20 (3%) | 273.66 |  | 86.00 |  |
| Indigenous/ Pacific Islander | 4 (1%) | 26.00 |  | 38.00 |  |
| Unknown/ Other | 9 (2%) | 461.33 |  | 131.00 |  |
| Missing | 11 (2%) | 133.67 |  | 142.00 |  |
| **Age at death** | | | | | |
| 18-30 | 122 (21%) | 207.21 | 0.05 (0.98) | 40.50 | **8.81 (0.03)** |
| 31-44 | 143 (25%) | 224.37 |  | 22.00 |  |
| 45-54 | 138 (24%) | 204.25 |  | 38.00 |  |
| 55 and over | 174 (30%) | 222.96 |  | 60.50 |  |
| Missing | 4 (<1%) | 80.67 |  | 51.00 |  |
| **Custody status** | | | | | |
| Unconvicted | 412 (71%) | 169.43 | 3.31 (0.07) | 25.00 | **38850 (<0.0001)** |
| Convicted | 138 (24%) | 235.48 |  | 121.50 |  |
| Missing | 31 (5%) | 1141.94 |  | 54.00 |  |
| **Death place** | | | | | |
| Jail | 267 (47%) | 193.12 | 0.59 (0.56) | 19.00 | **23.20 (<0.0001)** |
| Hospital | 288 (50%) | 239.23 |  | 63.00 |  |
| Other | 2 (<1%) | 45.50 |  | 45.50 |  |
| Missing | 24 (3%) | 173.95 |  | 50.00 |  |
| **Cause of death** | | | | | |
| Natural | 275 (48%) | 228.83 | 1.55 (0.19) | 64.50 | **31.81 (<0.0001)** |
| Accident | 120 (21%) | 304.09 |  | 28.50 |  |
| Suicide | 93 (16%) | 154.00 |  | 11.50 |  |
| Homicide | 29 (11%) | 253.13 |  | 97.00 |  |
| Undetermined | 38 (7%) | 92.63 |  | 20.50 |  |
| Missing | 25 (3%) | 10.94 |  | 1.79 |  |
| **COVID-rel. death** | | | | | |
| Yes | 22 (5%) | 452.21 | 3.54 (0.06) | 150.50 | **3029 (<0.0001)** |
| No | 543 (93%) | 209.21 |  | 37.00 |  |
| Missing | 16 (2%) | 87.73 |  | 49.00 |  |
| **Acute-substance-related death** | | | | | |
| Yes | 79 (14%) | 394.08 | **9.81 (0.002)** | 18.00 | 20256 (0.21) |
| No | 500 (85%) | 187.25 |  | 41.00 |  |
| Missing | 2 (<1%) | 51.00 |  | 51.00 |  |
| **Substance-related type**^†^ | | | | | |
| Acute (overdose) | 79 (14%) | 394.08 | 1.39  (0.24) | 18.00 | **14.50 (<0.0001)** |
| Chronic | 53 (9%) | 127.35 |  | 11.00 |  |
| Potentially chronic | 4 (<1%) | 209.59 |  | 8.48 |  |
| Undetermined | 18 (3%) | 92.99 |  | 18.50 |  |
| Traumatic w/ substances | 9 (2%) | 31.40 |  | 2.36 |  |
| Adverse effects of drugs | 2 (<1%) | 83.00 |  | 83.00 |  |
| Not substance related | 414 (71%) | 202.69 |  | 50.00 |  |
| Missing | 2 (<1%) | 51.00 |  | 51.00 |  |

Missing values excluded in statistical tests

^*^ Variables collapsed into Black, Latino, White, Other for statistical tests

^†^ Variables collapsed into substance-related and not substance-related for statistical tests

**References**

[**1.**](https://www.zotero.org/google-docs/?AOuArw) [Million Dollar Hoods. Data Dictionary Los Angeles Police Department 2010-2020. https://ucla.app.box.com/s/qnnluoo4bb89he59sfz7vwbgwf36b0vz.](https://www.zotero.org/google-docs/?AOuArw)

[2.](https://www.zotero.org/google-docs/?AOuArw) [Carson, E. A. *Mortality in Local Jails, 2000–2019 – Statistical Tables*. https://bjs.ojp.gov/content/pub/pdf/mlj0019st.pdf (2021).](https://www.zotero.org/google-docs/?AOuArw)
